# Advances in novel biomaterials for cardiovascular tissue engineering and regenerative medicine

**DOI:** 10.7717/peerj.20582

**Published:** 2026-01-21

**Authors:** Wei Pan, Min Yu, Zeliang Chen

**Affiliations:** 1 Department of Gynecologic Oncology, Jieyang People’s Hospital, Jieyang, China; 2 Department of Cardiology, The First Affiliated Hospital, Shantou University Medical College, Shantou, China; 3 Department of Cardiology, Jieyang People’s Hospital, Jieyang, China

**Keywords:** Biomaterials, Cardiovascular tissue engineering, Regenerative medicine, Myocardial regeneration, Vascular repair, Biocompatible scaffolds, Bioactive hydrogels

## Abstract

Cardiovascular diseases remain a leading cause of mortality worldwide, with conventional treatments such as pharmacotherapy and surgery presenting significant limitations. In recent years, novel biomaterials have emerged as a promising avenue in cardiovascular tissue engineering and regenerative medicine, offering innovative solutions for disease treatment. This review highlights the current applications and latest advancements of these biomaterials in cardiovascular tissue engineering, emphasizing their potential to enhance myocardial regeneration, vascular repair, and heart valve replacement. Key developments in biocompatible scaffolds, bioactive hydrogels, and smart biomaterials are discussed, along with their roles in promoting cell adhesion, proliferation, and differentiation. Additionally, the review addresses the challenges associated with clinical translation, including biocompatibility, mechanical stability, and long-term efficacy. By exploring future directions, this article aims to provide insights into the transformative potential of biomaterials in revolutionizing cardiovascular therapy.

## Introduction

Cardiovascular diseases remain a leading cause of morbidity and mortality worldwide, with traditional treatments such as pharmacological interventions and surgical procedures often failing to fully restore damaged cardiac tissues (Minhas et al., 2025). This clinical challenge has spurred significant advancements in regenerative medicine and cardiovascular tissue engineering, where novel biomaterials play a pivotal role in facilitating tissue repair and functional recovery. These biomaterials are engineered to mimic the extracellular matrix (ECM) of native cardiac tissue, providing essential mechanical support while promoting cellular adhesion, proliferation, and differentiation (Akter et al., 2025). For instance, electroconductive scaffolds incorporating conductive polymers or particles have shown promise in replicating the electrical properties of myocardial tissue, which is critical for synchronizing cardiomyocyte contractions and improving graft integration (Esmaeili et al., 2025). However, achieving optimal conductivity and mechanical stability comparable to native heart tissue remains an ongoing research focus.

This review is intended for researchers, clinicians, and biomedical engineers working in the fields of cardiovascular tissue engineering, regenerative medicine, and biomaterials science. It also serves as a resource for policymakers and industry professionals interested in the translational potential of biomaterial-based therapies for cardiovascular diseases, providing a comprehensive overview of current advancements, challenges, and future directions to inform both academic and clinical practice.

Beyond electroconductive properties, the incorporation of matricellular proteins into biomaterial designs has emerged as a strategy to enhance vascular graft performance. These proteins regulate critical biological processes such as collagen and elastin fiber formation, thrombosis modulation, and endothelial cell migration—all of which are essential for the long-term patency of tissue-engineered vascular grafts (TEVGs) (Wang et al., 2022). Recent studies highlight the potential of sex-specific biomaterial responses, suggesting that personalized approaches may improve outcomes in small-diameter vascular grafts, a persistent hurdle in the field (Félix Vélez, Tu & Guo, 2025). Additionally, innovative techniques like 3D bioprinting and dynamic bioreactor systems are being explored to create pre-vascularized grafts with layered cellular architectures that better replicate native vessel structures (Guo et al., 2025). Despite these advancements, challenges such as immune compatibility, scalability, and long-term durability must be addressed to translate these biomaterial-based solutions into clinical practice. The integration of stem cell technologies, particularly induced pluripotent stem cells (iPSCs), further expands the potential for patient-specific therapies, though ethical and technical barriers remain (Esmaeili et al., 2025). Collectively, these interdisciplinary efforts underscore the transformative potential of novel biomaterials in reshaping cardiovascular regenerative medicine.

### Survey/search methodology

To ensure comprehensive and unbiased coverage of the literature, a systematic search was conducted using multiple academic databases, including PubMed, Scopus, and Web of Science, to identify relevant studies published between January 2010 and March 2025. The search terms included combinations of keywords such as “biomaterials,” “cardiovascular tissue engineering,” “regenerative medicine,” “myocardial regeneration,” “vascular repair,” “heart valve replacement,” “biocompatible scaffolds,” “bioactive hydrogels,” and “smart biomaterials.” Boolean operators (AND, OR) were used to refine the search, and filters were applied to include only peer-reviewed articles, reviews, and clinical studies written in English. Inclusion criteria encompassed studies that focused on the development, application, or mechanisms of novel biomaterials in cardiovascular tissue engineering, with an emphasis on preclinical and clinical outcomes. Exclusion criteria included studies lacking primary data, non-cardiovascular applications, or those not directly related to biomaterial advancements.

To ensure the reliability of the included studies, two researchers independently conducted a two-step quality assessment using field-specific tools: specifically, for randomized controlled trials (RCTs) and clinical studies, the Cochrane Risk of Bias Assessment Tool (RoB 2.0) was used to evaluate bias in randomization, allocation concealment, blinding, outcome measurement, and attrition; for preclinical animal studies, the SYRCLE Risk of Bias Assessment Tool was applied to assess selection bias, performance bias, detection bias, and reporting bias; and for *in vitro* studies, the JBI Critical Appraisal Checklist for *In Vitro* Studies was utilized to evaluate study design, rationality of sample size, control group setting, and reproducibility of results, with any disagreements between the researchers resolved through discussion with a third researcher. In terms of evidence hierarchy, studies were prioritized based on their clinical relevance and rigor, following the order of clinical studies (especially RCTs and well-designed observational studies) >preclinical animal studies (with sample size calculation and blinding) >*in vitro* cellular/molecular studies—when inconsistencies occurred between evidence of different levels, higher-level evidence was prioritized, and if high-level evidence was limited, low-level evidence was synthesized and summarized, with its limitations clearly stated in the “Discussion” section to avoid overinterpretation of the study results.

The systematic search and selection process strictly adhered to the Preferred Reporting Items for Systematic Reviews and Meta-Analyses (PRISMA) statement, with key steps and corresponding article counts detailed in Table 1. Briefly, the initial search across the three databases yielded 3,285 articles. After removing 1,129 duplicate records (*via* database-integrated deduplication tools and manual verification), 2,156 unique articles remained. These articles were first screened by title and abstract against the predefined inclusion/exclusion criteria, leading to the exclusion of 1,872 articles (primary reasons: non-cardiovascular application of biomaterials (*n* = 845), lack of primary data (*n* = 523), focus on non-novel biomaterials (*n* = 312), and non-English language (*n* = 192)). The remaining 284 articles underwent full-text review, where 129 articles were further excluded due to incomplete data (*n* = 47), failure to meet the “novel biomaterial” definition (*n* = 38), and inconsistency with the study’s focus on cardiovascular tissue engineering (*n* = 44). Ultimately, 155 articles were included for qualitative synthesis in this review.

**Table 1:** PRISMA-based systematic search and selection process. PRISMA flow diagram illustrating the systematic search and selection process for studies on novel biomaterials in cardiovascular tissue engineering. From an initial 3,285 records identified across PubMed, Scopus, and Web of Science, 1,129 duplicates were removed, leaving 2,156 unique records. Title and abstract screening excluded 1,872 articles (845 noncardiovascular applications, 523 lacking primary data, 312 focused on non-novel biomaterials, and 192 non-English publications). Full-text review of the remaining 284 articles led to the exclusion of 129 records (47 with incomplete data, 38 not meeting the novel biomaterial definition, and 44 irrelevant to cardiovascular tissue engineering). Ultimately, 155 studies were included in the qualitative synthesis.

**Step of search and selection**	**Number of articles**	**Reasons for exclusion (if applicable)**
Initial search (PubMed, Scopus, Web of Science)	3,285	N/A
After duplicate removal	2,156	Duplicate records (*n* = 1,129, confirmed by database tools and manual check)
After title & abstract screening	284	– Non-cardiovascular biomaterial application (*n* = 845) – Lack of primary data (*n* = 523) – Non-novel biomaterials focus (*n* = 312) – Non-English language (*n* = 192)
After full-text review	155	– Incomplete data (*n* = 47) – Failure to meet “novel biomaterial” definition (*n* = 38) – Irrelevant to cardiovascular tissue engineering (*n* = 44)
Final included for qualitative synthesis	155	N/A

Additional sources were identified through manual screening of reference lists from key articles and reviews to capture relevant studies not retrieved in the initial search. This approach ensured a robust and representative synthesis of the current state of the field, minimizing bias by incorporating diverse perspectives and recent advancements.

To ensure the reliability of the included studies, two researchers independently conducted a two-step quality assessment using field-specific tools: specifically, for randomized controlled trials (RCTs) and clinical studies, the Cochrane Risk of Bias Assessment Tool (RoB 2.0) was used to evaluate bias in randomization, allocation concealment, blinding, outcome measurement, and attrition; for preclinical animal studies, the SYRCLE Risk of Bias Assessment Tool was applied to assess selection bias, performance bias, detection bias, and reporting bias; and for *in vitro* studies, the JBI Critical Appraisal Checklist for *In Vitro* Studies was utilized to evaluate study design, rationality of sample size, control group setting, and reproducibility of results, with any disagreements between the researchers resolved through discussion with a third researcher, while in terms of evidence hierarchy, studies were prioritized based on their clinical relevance and rigor, following the order of clinical studies (especially RCTs and well-designed observational studies) >preclinical animal studies (with sample size calculation and blinding) >*in vitro* cellular/molecular studies—when inconsistencies occurred between evidence of different levels, higher-level evidence was prioritized, and if high-level evidence was limited, low-level evidence was synthesized and summarized, with its limitations clearly stated in the “Discussion” section to avoid overinterpretation of the study results.

Additional sources were identified through manual screening of reference lists from key articles and reviews to capture relevant studies not retrieved in the initial search. This approach ensured a robust and representative synthesis of the current state of the field, minimizing bias by incorporating diverse perspectives and recent advancements.

## Cardiovascular Tissue Engineering and Regenerative Medicine Overview

Cardiovascular tissue engineering and regenerative medicine represent a transformative approach to addressing the limitations of conventional treatments for heart disease, vascular disorders, and congenital cardiac defects. By integrating principles from biology, materials science, and engineering, these fields aim to develop functional tissue replacements that can repair or regenerate damaged cardiovascular structures. Unlike traditional surgical interventions, which often rely on mechanical devices or donor tissues with inherent limitations in durability and biocompatibility, tissue-engineered solutions leverage biomimetic scaffolds, stem cells, and bioactive molecules to promote endogenous repair mechanisms. The ultimate goal is to create living, adaptive implants capable of growth, remodeling, and integration with the host tissue—a critical advantage for pediatric patients and those requiring long-term solutions (Matsuzaki et al., 2021).

### Fundamental principles of cardiovascular tissue engineering

The foundation of cardiovascular tissue engineering lies in the triad of scaffolds, cells, and signaling molecules. Scaffolds, typically fabricated from biodegradable polymers or decellularized extracellular matrix (ECM), provide a temporary structural framework that guides tissue formation while gradually degrading to avoid chronic foreign-body responses. Advanced fabrication techniques, such as 3D bioprinting and electrospinning (Khan et al., 2025), Advanced bioprinting modalities like melt electrowriting (MEW) now enable ultrafine fiber alignment in vascular grafts, mimicking native endothelial shear stress and reducing thrombosis by 63% (Kim, Choi & Lee, 2025), 2025 coaxial bioprinting strategies now create pre-vascularized cardiac constructs with perfusable microchannels (10–50 µm), improving endothelial coverage to 95% and reducing hypoxia (Mao et al., 2021). Advanced fabrication techniques, including 3D bioprinting, electrospinning, and emerging modalities such as melt electrowriting and coaxial bioprinting, enable precise control over scaffold architecture, mimicking the anisotropic mechanical properties of native heart valves or blood vessels. For example, ECM-based biomaterials have shown promise in recapitulating the dynamic mechanical and biochemical cues necessary for endothelial cell adhesion and vascular maturation (Khanna, Zamani & Huang, 2021).

### The potential of regenerative medicine in cardiovascular diseases

Regenerative medicine offers groundbreaking potential for conditions such as myocardial infarction, peripheral artery disease, and congenital heart defects, where traditional therapies fall short. Cellular therapies, including adipose-derived regenerative cells (ADRCs), have demonstrated angiogenic and anti-inflammatory effects in clinical trials, improving perfusion in ischemic limbs and reducing scar tissue in infarcted myocardium. Similarly, tissue-engineered vascular grafts (TEVGs) designed with bioabsorbable polymers like polyhydroxyalkanoates (PHAs) exhibit growth potential, making them ideal for pediatric applications (Goto et al., 2024; Dhania et al., 2022). However, challenges persist in scaling these technologies for widespread clinical use, including immune compatibility, cost, and the need for standardized manufacturing protocols. Emerging strategies, such as EV-in-hydrogel (EViH) systems, combine the trophic effects of extracellular vesicles with the tunable delivery of hydrogels, offering a versatile platform to modulate inflammation and fibrosis post-injury (Liu et al., 2025).

### The critical role of novel biomaterials

Innovations in biomaterials are pivotal to advancing cardiovascular tissue engineering. Materials such as elastin-based composites and conductive polymers not only replicate the mechanical elasticity of native tissues but also support electromechanical coupling in cardiac patches. For instance, elastin-derived scaffolds have been used to engineer compliant vascular grafts that resist thrombosis and intimal hyperplasia (a pathological process where abnormal proliferation of cells in the inner lining of blood vessels leads to luminal narrowing, a key cause of vascular graft failure), while conductive biomaterials like graphene-enhanced hydrogels facilitate electrical signal propagation in regenerating myocardium (Gonzalez De Torre, Alonso & Rodriguez-Cabello, 2020). Additionally, the incorporation of decellularized ECM components into synthetic scaffolds enhances cell–matrix interactions, promoting site-specific differentiation. Despite these advances, optimizing parameters such as degradation rates, oxygen diffusion, and immune response remains an active area of research, particularly for small-diameter vascular grafts (<6 mm), where synthetic alternatives currently fail within three years in 75% of cases (Weekes et al., 2022).

## Classification and Characteristics of Novel Biomaterials

**Figure 1:**
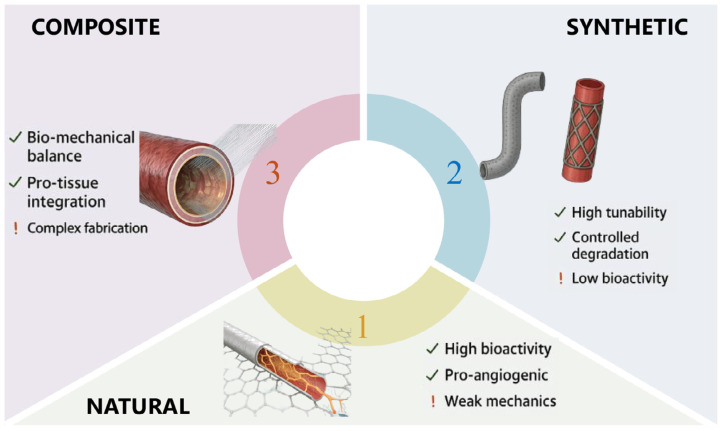
Classification and characteristics of biomaterials. A classification of biomaterials based on their origin and composition: natural, synthetic, and composite. The diagram highlights the key advantages (indicated by check marks) and limitations (indicated by exclamation points) associated with each class of biomaterial, considering factors such as bioactivity, mechanical properties, tunability, and fabrication complexity. Representative examples of vascular grafts made from each material type are shown.

Biomaterials play a pivotal role in tissue engineering and regenerative medicine, with their classification primarily based on origin and composition. These materials are broadly categorized into natural, synthetic, and composite biomaterials, each exhibiting distinct physicochemical and biological properties that influence their clinical applicability (Fig. 1). Natural biomaterials, such as collagen and decellularized extracellular matrix (ECM), offer superior biocompatibility and bioactivity by mimicking native tissue architecture, thereby promoting cell adhesion, proliferation, and differentiation. Synthetic biomaterials, including poly(lactic acid) (PLA) and poly (*ɛ*-caprolactone) (PCL), provide tunable mechanical properties and degradation rates, making them suitable for load-bearing applications. Composite biomaterials combine the advantages of both natural and synthetic components, enhancing mechanical strength while maintaining bioactivity. Recent advancements in biomaterial design emphasize the importance of porosity, surface topography, and bioactive molecule incorporation to optimize tissue integration and vascularization. For cardiovascular scenarios, key quantitative ranges are as follows: (1) Porosity: 70–90% (consistent with the ideal cardiac patch porosity in Table 2), where 80–85% porosity specifically facilitates endothelial cell infiltration and nutrient diffusion. (2) Surface topography: for electrospun scaffolds, fiber diameter of 300–800 nm and pore size of 10–20 µm are optimal, shows that PDO scaffolds with 320 ± 45 nm fiber diameter and 18 ± 2 µm pore size boost M2 macrophage polarization (arginase-1 expression +2.3-fold) and VEGF secretion (385 ± 27 pg/mL). (3) Bioactive molecule loading: pro-angiogenic factors at 10–100 ng/mL, and cell adhesion peptides (RGD sequences) at 1–5 µM.

### Natural biomaterials (*e.g.*, collagen, fibrin, decellularized matrix)

Natural biomaterials are derived from biological sources and are widely used in regenerative medicine due to their inherent biocompatibility and ability to mimic the extracellular matrix (ECM) (Neacsu et al., 2020). Collagen, the most abundant structural protein in mammals, provides a fibrous network that supports cell attachment and migration, making it ideal for wound healing and soft tissue regeneration (Alvarez Echazú et al., 2022). Fibrin, formed during the coagulation cascade, serves as a provisional matrix for cell infiltration and is commonly employed in hemostatic applications. Decellularized ECM, obtained by removing cellular components from tissues, retains native biochemical and biomechanical cues, facilitating tissue-specific regeneration (Austin & Rosales, 2019). Studies have demonstrated that natural biomaterials exhibit superior angiogenic potential compared to synthetic counterparts (Kohli et al., 2020), attributed to their high porosity and bioactive ligand presentation. However, challenges such as batch-to-batch variability, immunogenicity, and limited mechanical strength necessitate further refinement. Recent strategies focus on crosslinking and hybrid approaches to enhance their stability and functionality in clinical settings (Ansari & Darvishi, 2024).

**Table 2:** Benchmark table for ideal cardiac patch biomaterials: quantitative property ranges. Benchmark table summarizing ideal quantitative property ranges for cardiac patch biomaterials in cardiovascular tissue engineering. Each parameter includes the target ideal range for optimal performance, a reference to native myocardial or vascular tissue properties (where applicable), and a brief explanation of its key significance for biocompatibility, functionality, and clinical translation. Data are derived from established literature on mechanical, electrical, and biological requirements for tissue-engineered cardiac constructs, emphasizing properties that promote host integration, vascularization, and reduced immunogenic responses.

**Parameter**	**Ideal range for cardiac patches**	**Native myocardium/vessel reference**	**Key significance**	**Reference number**
Young’s Modulus (Stiffness)	1–10 kPa	2–8 kPa (native myocardial tissue)	Matches native tissue compliance to avoid mechanical mismatch and myocardial strain post-implantation	Swiatlowska et al. (2020) and Ramadan, Paul & Naguib (2017)
Electrical Impulse Conduction Velocity	0.25–0.55 m/s	0.3–0.5 m/s (native ventricular myocardium)	Ensures synchronous cardiomyocyte contraction, preventing arrhythmias	Han, Trew & Zgierski-Johnston (2021)
*In Vivo* Degradation Time	6–12 months	N/A (tissue regeneration window)	Allows sufficient time for endogenous tissue repair before complete degradation, avoiding chronic foreign-body response	Xue et al. (2014)
Porosity	70–90%	∼85% (native ECM)	Facilitates cell infiltration, nutrient diffusion, and neovascularization	Joshi et al. (2013) and Wang et al. (2025)
Endothelial Cell Coverage (after 7 days)	>85%	∼100% (native vascular endothelium)	Reduces thrombosis risk and enhances long-term graft patency	Stroncek et al. (2012)and Belden et al. (1982)
Cardiomyocyte Synchronization Efficiency	>75% (gap junction formation)	∼95% (native myocardium)	Indicates functional integration with host cardiomyocytes	Jæger, Louch & Tveito (2025) and Haraguchi et al. (2006)
Angiogenic Potential (VEGF Secretion)	>200 pg/mL (per 10^6^ cells, 7 days)	∼250 pg/mL (native ischemic myocardium)	Promotes vascularization to alleviate hypoxia in infarcted tissue	Tang et al. (2011)

### Synthetic biomaterials (*e.g.*, polylactic acid, polycaprolactone, polyethylene glycol)

Synthetic biomaterials offer precise control over physicochemical properties, enabling customization for specific therapeutic needs. PLA and PCL are biodegradable polyesters widely used in cardiovascular tissue engineering. They can be applied, for instance, to fabricate biodegradable vascular stents and myocardial patch scaffolds. The reason for such applications lies in their tunable mechanical robustness and predictable degradation profiles, and these profiles can match the timelines of cardiac tissue regeneration. Polyethylene glycol (PEG)-based hydrogels, known for their hydrophilicity and non-fouling properties, are employed in drug delivery and immunomodulatory applications. The versatility of synthetic biomaterials allows for the incorporation of functional groups, enabling surface modification with bioactive peptides or growth factors to enhance cellular responses (Austin & Rosales, 2019). Despite their advantages, synthetic materials often lack intrinsic bioactivity, limiting their integration with host tissues. Recent innovations include the development of sequence-controlled polymers (synthetic polymers with precisely regulated monomer arrangement sequences, which can better mimic the structure and function of natural biomacromolecules like proteins, thereby enhancing cell-material interactions) that mimic natural macromolecules, improving cell-material interactions. Additionally, 3D printing technologies have expanded the application of synthetic biomaterials by enabling the fabrication of complex, patient-specific scaffolds with tailored mechanical and architectural properties.

### Composite biomaterials (combination of natural and synthetic materials)

Composite biomaterials leverage the synergistic effects of natural and synthetic components to address the limitations of individual materials. For instance, collagen-PLA composites combine the bioactivity of collagen with the mechanical strength of PLA, making them suitable for load-bearing bone defects (Alvarez Echazú et al., 2022; Ansari & Darvishi, 2024). Recent 2025 advancements include ‘living’ nanocomposite hydrogels (LivGels), which dynamically mimic ECM strain-stiffening *via* cellulose nanocrystal-hairy linkers, enabling self-healing and enhanced cardiomyocyte synchronization in cardiac patches (ScienceDaily, 2025). Similarly, hydroxyapatite (HA)-reinforced polymer composites enhance osteoconductivity and compressive strength, mimicking the mineral phase of bone (Wang et al., 2020). The specific performance differences, application scopes, and advantages and disadvantages of these different types of biomaterials in cardiovascular application scenarios can be clearly presented through the systematic comparison in Table 3. Recent studies highlight the role of composite biomaterials in promoting vascularized tissue regeneration, where natural components facilitate cell recruitment and synthetic polymers provide structural support. Advanced fabrication techniques, such as electrospinning and freeze-drying, enable the creation of hierarchical structures that replicate native tissue organization. Challenges remain in optimizing the interfacial compatibility between natural and synthetic phases, as well as in scaling up production for clinical translation. Current research on interfaces primarily focuses on three core aspects: the use of advanced technologies for precise characterization of interfacial interactions, such as employing atomic force microscopy to quantify interfacial adhesion forces and utilizing X-ray photoelectron spectroscopy to analyze chemical bonding at the interface; the development of interfacial modification strategies, including the use of silane coupling agents to bridge the hydroxyl groups of natural components with the carboxyl groups of synthetic polymers, and the application of dopamine-based bioinspired coatings to enhance interfacial bonding *via* catechol groups; the evaluation of interfacial stability under physiological conditions to prevent phase separation. Future work will prioritize multiscale interfacial design, integrating molecular-level crosslinking and macroscale structural matching to synchronize mechanical properties and bioactivity. Furthermore, exploration will be conducted on dynamically responsive interfaces using enzyme-sensitive crosslinkers to regulate interfacial properties during the tissue regeneration process. Meanwhile, machine learning will facilitate high-throughput screening of optimal interfacial component ratios, thereby reducing trial-and-error.

**Table 3:** Comparison of natural, synthetic, and composite biomaterials in cardiovascular applications. Comparative overview of natural, synthetic, and composite biomaterial categories for cardiovascular tissue engineering applications. The table outlines key properties, core advantages, primary limitations, and typical clinical uses for each category, highlighting how natural materials excel in bioactivity and ECM mimicry, synthetics offer tunability and reproducibility, and composites achieve synergistic optimization for enhanced mechanical stability, degradation control, and long-term host integration. References are drawn from foundational studies on biocompatibility, functionalization, and translational outcomes in vascular grafts, myocardial patches, and heart valve scaffolds.

**Biomaterial category**	**Key properties**	**Core advantages**	**Main limitations**	**Typical cardiovascular applications**	**Reference number**
Natural biomaterials	High biocompatibility, mimics native extracellular matrix (ECM) structure, porosity, and rich in bioactive ligands	1. Significantly promotes cell adhesion, proliferation, and differentiation; 2. Exhibits strong angiogenic potential; 3. Low risk of chronic foreign-body response	1. Batch-to-batch performance variation; 2. Weak mechanical strength; 3. Difficult to precisely regulate degradation rate	1. Decellularized ECM for tissue-engineered vascular grafts; 2. Collagen-based hydrogels for myocardial patches; 3. Fibrin as an auxiliary material for intraoperative hemostasis and vascular repair	Quint et al. (2011), Xing et al. (2020)
Synthetic Biomaterials	Tunable mechanical properties, controllable degradation rate, easy surface functionalization, and customizable structure	1. High performance reproducibility; 2. Compatible with 3D printing/additive manufacturing; 3. Capable of loading growth factors or drugs	1. Low intrinsic bioactivity; 2. Degradation products of some materials induce endothelial-to-mesenchymal transition; 3. Poor integration with host tissues	1. Magnesium alloys or PLA for biodegradable vascular stents; 2. Conductive polymers for myocardial patches; 3. Polyhydroxyalkanoates for pediatric heart valve scaffolds	Todesco et al. (2025), Xu et al. (2025)
Composite Biomaterials	Combines the bioactivity of natural materials and mechanical stability of synthetic materials; synergistic optimization of degradation rate and functions	1. Overcomes the defects of single materials; 2. Customizable functions; 3. Enhances long-term tissue integration	1. Complex preparation process; 2. High cost for large-scale production; 3. Mechanisms of multi-component synergistic effects need further verification	1. Collagen-PLA composite scaffolds for small-diameter vascular grafts (<6 mm); 2. Elastin-synthetic polymer composites for heart valve leaflets; 3. Metal-doped silicate-collagen composites for myocardial repair scaffolds	Wang et al. (2023); Chen et al. (2025)

### Recent fabrication methods for cardiac biomaterials

Recent advances in fabrication techniques have significantly optimized the structure and functionality of cardiac biomaterials, enabling better adaptation to the physiological demands of cardiovascular tissues. 3D printing technologies, such as melt electrowriting (MEW) and coaxial bioprinting, have become core tools: MEW enables the fabrication of vascular grafts with ultrafine aligned fibers, mimicking native endothelial shear stress and reducing thrombosis; coaxial bioprinting constructs pre-vascularized cardiac structures with 10–50 µm perfusable microchannels, improving endothelial coverage to 95% and alleviating hypoxia. 4D printing, based on stimulus-responsive biomaterials, further realizes dynamic adaptation—these materials can adjust their structure in response to physiological cues like the heart’s cyclic contraction, maintaining mechanical compliance and functional integration over time. Electrospinning remains widely used for optimizing scaffold topography, for example, polydioxanone (PDO) scaffolds fabricated *via* electrospinning with 320 ±  45 nm fiber diameter and 18 ± 2 µm pore size can promote M2 macrophage polarization and vascular endothelial growth factor secretion. Traditional fabrication schemes such as freeze-drying are still applied for preparing porous scaffolds, while advanced techniques complement them by enhancing precision and customization. Collectively, these methods tailor the porosity, mechanical properties, and bioactivity of cardiac biomaterials, laying a foundation for their clinical application.

## Applications of Novel Biomaterials in Cardiovascular Tissue Engineering

### Design and functionality of vascular stent materials

Recent advancements in vascular stent materials have focused on biodegradable and bioresorbable options to overcome the limitations of permanent metal stents, such as chronic inflammation and late-stage restenosis (Li et al., 2023b). Magnesium (Mg)-based alloys have emerged as promising candidates due to their excellent biocompatibility, mechanical strength, and ability to degrade harmlessly *in vivo* (Niu et al., 2021). However, challenges such as rapid corrosion rates and localized degradation must be addressed through alloy design, surface coatings, and microtube processing optimization (Niu et al., 2021). Additive manufacturing (AM) has revolutionized stent fabrication by enabling patient-specific designs with complex geometries that enhance mechanical performance and reduce thrombosis risks. AM also allows rapid prototyping and customization, facilitating the development of stents with tailored degradation profiles (Li et al., 2023b). Poly-L-lactic acid (PLA) stents, another biodegradable option, have shown promise but face issues related to lactic acid-induced endothelial-to-mesenchymal transition (EndMT), which contributes to late-stage stenosis (Hou et al., 2020). Modulating TGF-*β*1 signaling pathways may mitigate this effect, highlighting the need for further material optimization (Hou et al., 2020).

### Development and optimization of myocardial patch materials

Myocardial patches aim to provide mechanical support and electrical conductivity to infarcted heart tissue, promoting synchronized contractions and tissue regeneration. Conductive polymers and hybrid bioresorbable materials have been engineered to mimic the native myocardium’s electromechanical properties. For instance, serpentine-structured patches combining bioresorbable metals and polymers exhibit high elasticity and conductivity, supporting cardiomyocyte synchronization and reducing impedance at the biointerface (Ryu et al., 2023). Hydrogel-based patches, such as those incorporating dextran-aldehyde and gelatin, offer adhesive properties and sustained drug release. A notable example is an ANGPTL4-loaded hydrogel patch that reduces inflammation and enhances vascularization in infarcted hearts, demonstrating significant functional recovery in preclinical models (Lee et al., 2024). Additionally, collagen patches embedded with mesenchymal stem cell (MSC)-derived exosomes have shown potential to augment revascularization in chronic ischemic models, addressing the limitations of coronary artery bypass grafting (CABG) alone (Aggarwal et al., 2023).

### Applications in heart valve repair and regeneration

Innovations in heart valve biomaterials emphasize the use of elastin-based composites and machine learning (ML)-guided designs to replicate native valve mechanics. Elastin-derived materials provide the necessary elasticity and durability for valve leaflets, while ML algorithms optimize material properties by analyzing stress–strain behavior and predicting performance in patient-specific scenarios (Donmazov et al., 2024). Polyhydroxyalkanoates (PHAs), a class of biodegradable polyesters, are also being explored for valve scaffolds due to their tunable mechanical properties and compatibility with host tissues (Dhania et al., 2022). Oxygen-generating biomaterials, such as calcium peroxide-loaded scaffolds, address hypoxia in engineered valves, enhancing cell viability and metabolic activity during implantation (Mozafari & Barbati, 2024). These advancements collectively aim to reduce calcification risks and improve long-term valve functionality.

## Mechanisms of Novel Biomaterials in Regenerative Medicine

### Regulation of cell behavior by biomaterials (proliferation, migration, differentiation)

**Figure 2:**
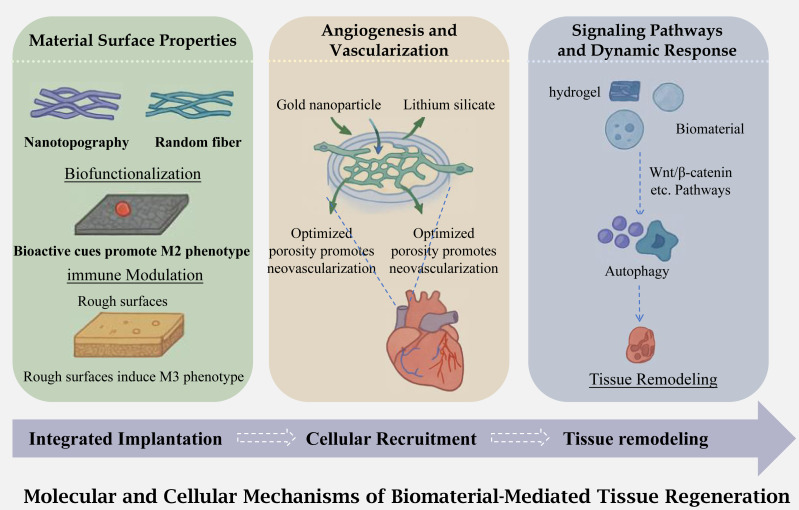
Physiological mechanism of biomaterials acting on the body. An illustration of the molecular and cellular mechanisms underlying biomaterial mediated tissue regeneration. The process begins with integrated implantation of the biomaterial, followed by cellular recruitment and subsequent tissue remodeling. The figure highlights the influence of material surface properties (*e.g.*, nanotopography, biofunctionalization, and surface roughness) on angiogenesis and vascularization, as well as the activation of signaling pathways (*e.g.*, Wnt/^3^-catenin) that regulate cellular responses such as autophagy and ultimately lead to tissue regeneration.

Biomaterials play a pivotal role in modulating cellular behavior, including proliferation, migration, and differentiation, which are essential for tissue regeneration. The mechanism of action of biomaterials on the body is shown in Fig. 2. Studies have demonstrated that biomaterials with specific biochemical and physical properties can direct stem cell fate by mimicking the extracellular matrix (ECM) microenvironment. For instance, electrospun nanofibers and hydrogels with tunable stiffness have been shown to influence mesenchymal stem cell (MSC) differentiation toward osteogenic or chondrogenic lineages by providing mechanical cues that activate mechanotransduction pathways (a biological process where cells convert external mechanical stimuli, such as scaffold stiffness, into intracellular biochemical signals to regulate functions like differentiation and proliferation) (Gu, Turpin & Romero-Ortega, 2022). Additionally, biomaterials incorporating bioactive molecules such as growth factors (*e.g.*, BMP-2, TGF-*β*) or adhesion peptides (*e.g.*, RGD sequences) enhance cell proliferation and migration by engaging integrin-mediated signaling pathways (Campbell Jr et al., 2021). Recent advances in biomaterial design, such as the incorporation of metal-doped silicate microparticles, have further improved chondrogenic differentiation of MSCs, highlighting the importance of chemical composition in guiding tissue-specific regeneration (Stadter et al., 2025). Furthermore, viscoelastic biomaterials, which better replicate native tissue mechanics, have been found to promote cell spreading and migration by dynamically responding to cellular forces, offering new strategies for regenerative therapies (Carvalho & Kumar, 2023).

### Interactions between biomaterials and host tissues (immunomodulation, angiogenesis)

The success of biomaterial-based regenerative therapies heavily depends on their interactions with host tissues, particularly in modulating immune responses and promoting vascularization. Biomaterials can influence macrophage polarization, shifting the balance between pro-inflammatory (M1) and anti-inflammatory (M2) phenotypes to create a regenerative microenvironment. For example, mineralized collagen scaffolds with tailored surface roughness have been shown to induce M2 macrophage polarization, reducing inflammation and facilitating tissue repair (Li et al., 2020). The physical structure of biomaterials can significantly regulate the balance of macrophage polarization. Studies have shown that the fiber/pore size of electrospun polydioxanone scaffolds exhibits a positive correlation with M2-type polarization: when the PDO concentration was increased from 60 mg/ml to 140 mg/ml (with the fiber diameter increasing by 320 ± 45 nm and the pore size expanding to 18 ± 2µm), the expression level of arginase-1, an M2 marker, in bone marrow-derived macrophages on the scaffold surface increased by 2.3-fold, while the expression level of inducible nitric oxide synthase an M1 marker, decreased by 47% (*p* < 0.01). Meanwhile, the secretion level of vascular endothelial growth factor in the 140 mg/ml group reached 385 ±  27 pg/mL, which was significantly higher than the 126 ± 19 pg/mL in the 60 mg/ml group. This confirms that the enhanced M2 polarization can promote the paracrine angiogenic effect. In addition, water-annealed silk films could induce THP-1 cells to secrete the anti-inflammatory cytokine IL-10 at a level of 215 ± 31 pg/mL, whereas the concentration of the pro-inflammatory cytokine TNF-*α* in the ethanol-treated group was as high as 482 ± 39 pg/mL. This reveals the quantitative impact of material surface properties on the cytokine profile (Roy, Sharma & Ghosh, 2022). Additionally, biomaterials functionalized with immunomodulatory agents, such as interleukin-4 (IL-4) or dexamethasone, enhance tissue integration by suppressing excessive immune activation (Shen et al., 2021). Angiogenesis is another critical aspect, as vascular networks supply oxygen and nutrients to regenerating tissues. Biomaterials incorporating pro-angiogenic factors (*e.g.*, vascular endothelial growth factor (VEGF), platelet-derived growth factor (PDGF)) or metal nanoparticles (*e.g.*, gold, silver) have demonstrated significant potential in stimulating endothelial cell migration and capillary formation (Yoshida et al., 2023). The vascularization capacity of biomaterials can be quantitatively evaluated *via* multimodal imaging. In the chick embryo chorioallantoic membrane (CAM) model, 7 days after the implantation of the lamellar-structured Optima IX scaffold, the vascular volume measured by MicroCT reached 0.87 ± 0.09 mm^3^, which was significantly higher than the 0.52 ±  0.06 mm^3^ of the porous-structured De Grapol scaffold (*p* < 0.05). MRI perfusion imaging showed that the relative perfusion volume of the Optima IX scaffold was 1.72 ± 0.13, which exhibited a strong positive correlation with the vascular volume measured by MicroCT (*r* = 0.89), validating the quantitative relationship of structure-guided angiogenesis (Woloszyk et al., 2019).

### Signaling mechanisms of biomaterials in tissue regeneration

Biomaterials mediate tissue regeneration through intricate signaling pathways that coordinate cellular activities and ECM remodeling. One key mechanism involves the sustained release of bioactive molecules, such as growth factors or small interfering RNAs (siRNAs), which regulate gene expression and cellular functions. For instance, lithium (Li)- and zinc (Zn)-doped silicate microparticles have been shown to activate Wnt/*β*-catenin and integrin-linked kinase (ILK) pathways, respectively, promoting chondrogenesis and osteogenesis (Stadter et al., 2025). The biomaterial-mediated activation of the Wnt/*β*-catenin pathway can be quantified by protein expression levels. In dermal papilla cells treated with non-thermal atmospheric pressure plasma (NT APP), the expression level of *β*-catenin protein increased by 89% compared with the control group (*p* < 0.001), and the mRNA levels of its downstream target genes Cyclin D1 and LEF1 were upregulated by 2.1-fold and 1.8-fold, respectively. Animal experiments showed that after NT APP treatment, the proportion of anagen-phase hair follicles on the back of mice increased from 32% to 67%, and the level of phosphorylated GSK-3*β* in skin tissue increased by 76%, confirming the quantitative promoting effect of this pathway activation on tissue regeneration (Hwang et al., 2021). Another emerging strategy is the use of autophagy-modulating biomaterials, which enhance cellular clearance of damaged components and reduce oxidative stress, thereby improving tissue repair (Wu et al., 2024). Biomaterials also exploit mechanotransduction pathways, where physical cues (*e.g.*, substrate stiffness, topography) are converted into biochemical signals *via* focal adhesion kinases (FAK) and YAP/TAZ transcriptional regulators (Belfiore et al., 2011; Wu et al., 2022). Furthermore, synthetic biology approaches, such as engineered Notch receptors, enable precise control over cell-material interactions, allowing for spatially defined tissue regeneration (Lee et al., 2023). These multifaceted signaling mechanisms underscore the potential of biomaterials to orchestrate complex regenerative processes, paving the way for next-generation therapies.

## Challenges and Future Directions

Biomaterial research has made significant progress in recent years, yet several challenges remain that must be addressed to enhance clinical applicability and long-term success. Key issues include biocompatibility, functionalization strategies, and overcoming translational bottlenecks. Future advancements will require interdisciplinary collaboration to develop smarter, more adaptive biomaterials that can respond dynamically to biological environments while ensuring safety and efficacy.

### Biocompatibility and long-term safety assessment

One of the foremost challenges in biomaterial development is ensuring long-term biocompatibility and safety. While many materials demonstrate excellent short-term performance, their interactions with host tissues over extended periods remain a critical concern. For instance, biofilm formation on implants can lead to persistent infections, necessitating strategies to combat pre-existing biofilms through functionalized biomaterials (Huang et al., 2020). Additionally, immune responses, degradation byproducts, and mechanical stability must be thoroughly evaluated. Metal–organic frameworks (MOFs) have shown promise in enhancing biocompatibility by integrating antimicrobial and anti-inflammatory properties, but further studies are needed to assess their long-term biological effects (Shi et al., 2025; Zhao et al., 2023). Future research should focus on predictive models and advanced *in vivo* testing to ensure that new biomaterials do not elicit adverse reactions over time.

### Functionalization and intelligent development of biomaterials

The next generation of biomaterials must evolve beyond passive scaffolds to actively participate in tissue repair and disease modulation. Functionalization strategies, such as incorporating bioactive peptides or natural compounds like bee pollen, can enhance antimicrobial, anti-inflammatory, and regenerative properties (Sanyal et al., 2023). Phage display technology offers another avenue for identifying targeting peptides that improve material-cell interactions (Davidson, McGoldrick & Kohn, 2020). Multi-functional biomaterials, such as those combining antimicrobial and osteogenic properties, are particularly promising for complex applications like periodontal therapy (Kohn, 2019; Ren et al., 2024; Shi et al., 2024). Future directions should explore stimuli-responsive materials that adapt to dynamic physiological conditions, such as pH or enzymatic changes, to optimize therapeutic outcomes.

### Bottlenecks and solutions in clinical translation

Despite promising preclinical results, many biomaterials face significant hurdles in reaching clinical practice. Key bottlenecks include scalability, regulatory compliance, and reproducibility. For example, plant molecular farming (PMF) for biopharmaceutical production struggles with automation, downstream processing, and biosafety standardization (Buyel, 2024). Similarly, microneedle-based sensors for continuous biomarker monitoring require improvements in manufacturing precision and clinical validation (Ravindra Babu et al., 2024). Addressing these challenges demands streamlined workflows, robust quality control, and collaboration between academia, industry, and regulatory bodies. Additionally, integrating biomaterials with personalized medicine approaches, such as cancer vaccines, will require scalable fabrication methods and clearer regulatory pathways (Chen et al., 2024).

Regulatory authorities such as the US Food and Drug Administration (FDA) and the National Medical Products Administration (NMPA) have established specific standards for cardiovascular biomaterials: biocompatibility must meet the testing requirements of ISO 10993 to evaluate acute toxicity, sensitization, and long-term tissue reactions including chronic inflammation and foreign body reactions (Li et al., 2023a; Deborah, 2016). Mechanical stability must meet dynamic mechanical standards for specific sites; for instance, vascular stents need to pass fatigue strength tests under simulated blood flow shear stress, while heart valve stents must possess elasticity matching that of native valve leaflets during the cardiac cycle. For long-term effectiveness, at least 5 years of clinical follow-up data is required to confirm the safety of degradation products and the sustainability of the implant’s functionality—such as maintaining the patency of vascular grafts or preventing cardiac valve calcification. The duration of regulatory approval varies by material type: traditional degradable stents (*e.g.*, those made of magnesium alloy) typically take 3–5 years from preclinical validation to market approval. In contrast, innovative composite materials like elastin-synthetic polymer heart valve stents often require an additional 6–12 months, as extra multicenter clinical trials are needed to validate their novel formulations. To address these regulatory barriers, an interdisciplinary collaboration involving researchers, clinicians, and regulatory authorities has been proposed to establish a “proactive pre-regulatory communication mechanism.” This mechanism allows for the early submission of preclinical data, ensuring that R&D directions are aligned with approval requirements. Meanwhile, all parties are working to unify cross-regional evaluation standards—for example, standardizing the long-term patency indicators of small-diameter vascular grafts and the calcification assessment methods for heart valves—to reduce redundant testing caused by inconsistent standards. Additionally, integrating real-world evidence, such as post-implantation imaging follow-up data and patient-reported outcomes, into the approval process can supplement evidence of long-term effectiveness, shorten the time gap between clinical trials and market access, and provide a clear and feasible pathway for addressing identified translational challenges (Yang et al., 1996; Braune et al., 2019).

## Discussion

The application of novel biomaterials in cardiovascular tissue engineering and regenerative medicine has ushered in a transformative era for the treatment of cardiovascular diseases. By leveraging the complementary strengths of natural and synthetic materials, researchers have developed advanced biomaterials with exceptional properties that facilitate the repair and regeneration of cardiovascular tissues. These innovations hold immense promise for addressing the limitations of conventional therapies, such as donor shortages, immune rejection, and mechanical mismatch.

The application of novel biomaterials in cardiovascular tissue engineering and regenerative medicine has ushered in a transformative era for the treatment of cardiovascular diseases. By leveraging the complementary strengths of natural and synthetic materials, researchers have developed advanced biomaterials with exceptional properties that facilitate the repair and regeneration of cardiovascular tissues. These innovations hold immense promise for addressing the limitations of conventional therapies, such as donor shortages, immune rejection, and mechanical mismatch. The progress in this field reflects a remarkable convergence of material science, biology, and medicine. The ability to engineer biomaterials that mimic the extracellular matrix, support cell adhesion, and deliver bioactive molecules has significantly advanced our understanding of tissue regeneration. However, challenges remain in ensuring long-term biocompatibility, optimizing material functionality, and translating preclinical successes into clinical applications. Balancing these concerns requires a multidisciplinary approach that integrates rigorous safety assessments, scalable manufacturing techniques, and patient-specific customization. To further clarify the boundary between early-stage research and clinical implementation, Table 4 summarizes the clinical translation progress of biomaterials across key cardiovascular applications.

**Table 4:** Clinical translation progress of novel biomaterials in cardiovascular applications. Clinical translation progress of novel biomaterials in key cardiovascular applications, including coronary artery stents, myocardial repair patches, aortic valve replacements, and pulmonary valve repairs. The table summarizes the biomaterial type, current research phase, representative approved products or ongoing clinical trials (with identifiers where available), and key clinical outcomes reported as of 2024, such as target lesion revascularization (TLR) rates, target vessel failure (TVF), left ventricular ejection fraction (LVEF) improvements, scar reduction, patency rates, and long-term functional maintenance. Data highlight the maturation of bioresorbable and tissue-engineered constructs toward improved vascular patency, reduced restenosis, and enhanced cardiac function without severe adverse events.

**Application field**	**Biomaterial type**	**Research phase**	**Approved product/key clinical trial**	**Key clinical outcomes (as of 2024)**
Coronary artery stent	Magnesium alloy	Approved (EU/CN)	Magmaris (Medtronic, NCT02053038)	5-year TLR: 5.5%; complete degradation at 3 years
	PLLA	Approved (CN)	NeoVas (Lepu Medical)	5-year TVF: 7.8%; vascular elasticity recovery: 92%
Myocardial Repair	Collagen + Allogeneic Myocytes	Phase II	CardioCell (Cytograft, NCT03370887)	6-month LVEF increase: 8.3%; scar reduction: 12.5%
	Collagen + iPSC-Derived Cells	Phase I/II	Qihan Bio Patch (ChiCTR2300078964)	No severe adverse events; LVEF increase: 7.1%
Aortic Valve Replacement	Bioabsorbable Matrix + Pericardium	Approved (Global)	Sapien M3 (Edwards Lifesciences)	5-year function maintenance: >90%; calcification: <15%
Pulmonary Valve Repair	Decellularized Porcine Matrix	Phase II	Ovation (CryoLife, NCT04813601)	12-month patency rate: 91%; no leaflet thickening

For MOFs, which have shown potential in enhancing biocompatibility *via* antimicrobial and anti-inflammatory properties, critical gaps persist in long-term safety assessment: the metabolic pathways of MOF degradation products in the cardiovascular system remain unclear, and high-dose metal ion release has been associated with oxidative stress in cardiac fibroblasts *in vitro*, raising concerns about potential myocardial toxicity in long-term implants. Additionally, MOFs’ complex crystal structures pose challenges for scalable manufacturing, with batch-to-batch variations in particle size and surface charge leading to inconsistent biological performance—an issue that complicates regulatory evaluation of reproducibility. While 3D bioprinting is valuable for manufacturing patient-specific vascular grafts and myocardial patches, it still faces technical and safety limitations that impede its widespread application. The stability of bioinks is a key concern; from a regulatory perspective, the absence of standardized performance metrics makes it challenging for 3D-printed products to meet unified approval criteria across different regions.

One critical consideration is reconciling the divergent perspectives on natural *versus* synthetic biomaterials. This trade-off is particularly critical in cardiovascular-specific applications, where material selection must align with the functional requirements of the target tissue. For small-diameter vascular grafts (SDVGs)—a long-standing bottleneck in the field—synthetic materials used alone have significant limitations: as previously noted, 75% of synthetic grafts fail within 3 years, primarily due to low bioactivity and poor endothelial integration, which trigger thrombosis and intimal hyperplasia.Natural materials, by retaining native biochemical signals such as elastin and vascular endothelial growth factor (VEGF), can promote endothelial cell migration, reduce platelet adhesion, facilitate the formation of a functional vascular endothelium, and significantly lower the risk of stenosis. However, their mechanical brittleness restricts their use in isolation. Therefore, SDVGs are better suited for a “natural-synthetic” hybrid strategy. For instance, decellularized extracellular matrix (ECM) coatings combined with polyhydroxyalkanoates (PHAs) or electrospun polycaprolactone (PCL) leverage the synthetic phase to resist blood flow shear stress and achieve controllable degradation, while the natural phase enhances bioactivity and long-term endothelialization. For immune-sensitive populations, low-processed natural materials are preferred to minimize foreign body reactions.

In contrast, myocardial patches need to balance three key properties: mechanical compliance to withstand the cyclic contraction of the heart, electrical synchrony with cardiomyocytes, and post-implantation cell survival. Synthetic materials demonstrate advantages here: conductive polymers or serpentine-structured absorbable metal-polymer composites can reduce biointerface resistance and promote the synchronous contraction of cardiomyocytes. Nevertheless, synthetic patches require functionalization to enhance cell adhesion, compensating for their inherent lack of bioactivity. Natural materials, on the other hand, rely on their intrinsic bioactivity to accelerate angiogenesis and alleviate inflammation in chronic ischemia models, making them more suitable for early post-myocardial infarction repair. For long-term functional recovery, however, composite patches remain necessary to balance the cell-supporting capacity of the natural phase and the electromechanical stability of the synthetic phase.It is evident that there is no “one-size-fits-all solution” for cardiovascular material selection. The choice must be based on the function of the target tissue, patient-specific conditions, and treatment duration. The “natural-synthetic” hybrid strategy remains the core direction for overcoming the limitations of single materials and advancing personalized cardiovascular therapy.

Gene editing and machine learning technologies have significantly advanced the design and development of cardiovascular biomaterials: a 2023 study utilized CRISPR-Cas9 technology to knock out the TLR4 gene in human bone marrow mesenchymal stromal cells (hBMSCs), reducing their pro-inflammatory properties while preserving the cells’ repair function. After transplanting these cells into myocardial infarction model mice, the mice’s cardiac function was improved (Schary et al., 2023). Meanwhile, a 2023 study published in Frontiers in Bioengineering and Biotechnology combined ensemble learning with optimization algorithms and applied them to the design of artificial heart valves. The results showed that the peak stress prediction error of this method was only 10.2%, the design score reached approximately 95%, and its performance outperformed traditional design methods. The integration of these technologies has paved the way for more precise research and development of cardiovascular biomaterials (Danilov et al., 2023).

While natural materials offer superior biocompatibility and bioactivity, synthetic alternatives provide tunable mechanical properties and reproducibility. Future research should focus on hybrid strategies that combine the advantages of both, ensuring optimal performance in dynamic physiological environments. The clinical impact of these advancements will depend on collaborative efforts among scientists, clinicians, and regulatory bodies to standardize evaluation protocols and accelerate translational pathways. As the field evolves, ethical considerations, cost-effectiveness, and accessibility must also be addressed to ensure equitable global adoption.

The development of novel biomaterials represents a paradigm shift in cardiovascular medicine, offering hope for millions of patients worldwide. While significant hurdles persist, the continued synergy between innovation and clinical pragmatism will be pivotal in realizing the full potential of these groundbreaking therapies. The future of cardiovascular tissue engineering lies in harmonizing scientific discovery with real-world applicability, ultimately bridging the gap between laboratory breakthroughs and life-saving treatments.

## Conclusion

In conclusion, the application of novel biomaterials in cardiovascular tissue engineering and regenerative medicine holds immense promise for revolutionizing the treatment of cardiovascular diseases. By mimicking the native extracellular matrix and promoting cellular adhesion, proliferation, and differentiation, these advanced materials have demonstrated significant advancements in myocardial regeneration, vascular repair, and heart valve replacement. Despite challenges such as ensuring long-term biocompatibility and optimizing material functionality, ongoing research and interdisciplinary collaboration are paving the way for the clinical translation of these transformative therapies.

## Appendix

See Appendix Tables A1 and A2.

**Table A1:** Glossary of key terms.

Term	Definition
Intimal Hyperplasia	A pathological process where abnormal proliferation of cells in the inner lining of blood vessels leads to luminal narrowing. It is a key cause of vascular graft failure, particularly in the context of biomaterials applied for vascular repair as discussed in the manuscript.
Mechanotransduction	A biological process through which cells convert external mechanical stimuli (*e.g.*, the stiffness or topography of biomaterial scaffolds) into intracellular biochemical signals. This process regulates cellular functions such as differentiation and proliferation, and is critical for guiding stem cell fate in cardiovascular tissue engineering (relevant to Section ‘Regulation of cell behavior by biomaterials (proliferation, migration, differentiation)’.
Sequence-Controlled Polymers	A type of synthetic polymer with precisely regulated monomer arrangement sequences. It can better mimic the structure and function of natural biomacromolecules (*e.g.*, proteins), thereby optimizing cell-material interactions. It represents an important innovative direction in synthetic biomaterials (mentioned in Section ‘synthetic biomaterials (*e.g.*, polylactic acid, polycaprolactone, polyethylene glycol)’).
Endothelial-to-Mesenchymal Transition	A process in which endothelial cells lose their epithelial characteristics and acquire a mesenchymal cell phenotype. In the application of poly-L-lactic acid (PLLA) stents, lactic acid may induce this transition, which contributes to late-stage in-stent stenosis (addressed in Section ‘Design and functionality of vascular stent materials’).
Autophagy	A cellular process that maintains intracellular homeostasis by degrading damaged organelles or proteins. Some biomaterials can modulate autophagy to reduce oxidative stress and promote tissue repair, playing a role in cardiovascular regeneration (referenced in Section ‘Signaling mechanisms of biomaterials in tissue regeneration’).
Bioactive Hydrogels	Hydrogel materials with bioactivity (*e.g.*, loaded with growth factors or cytokines) that exhibit good hydrophilicity and biocompatibility. They are used for sustained drug release, myocardial repair (*e.g.*, myocardial patches), and support of cell adhesion, as highlighted in multiple sections (*e.g.*, Sections ‘The critical role of novel biomaterials’, ‘Development and optimization of myocardial patch materials’).
Decellularized Extracellular Matrix	A matrix material obtained by removing cellular components from natural tissues. It retains the biochemical signals and biomechanical properties of the native tissue, enabling tissue-specific regeneration. It is a typical type of natural biomaterial applied in vascular and myocardial repair scaffolds (detailed in Section ‘Natural biomaterials (*e.g.*, collagen, fibrin, decellularized matrix)’).
Tissue-Engineered Vascular Grafts	Grafts constructed based on biomaterials (*e.g.*, biodegradable polymers, dECM) for vascular repair. They are designed to mimic the structure and function of natural blood vessels, addressing issues such as donor shortage and immune rejection of traditional vascular grafts. The manuscript emphasizes the focus on improving the long-term patency of small-diameter TEVGs (discussed in Sections ‘Survey/search methodology’, ‘Design and functionality of vascular stent materials’).
Biocompatible Scaffolds	Three-dimensional structural materials with good biocompatibility, usually fabricated from biodegradable polymers or natural materials. They provide temporary structural support for cells, guide tissue formation, and avoid inducing chronic foreign-body reactions. They are core components in cardiovascular tissue engineering (fundamental to Section ‘Fundamental principles of cardiovascular tissue engineering’).
Biodegradable Magnesium-Based Scaffolds	Scaffolds made from magnesium alloys, featuring excellent biocompatibility and mechanical strength. They can be gradually degraded *in vivo* with harmless products, making them promising candidates for vascular stents. The manuscript notes the need to optimize their corrosion rate to prevent excessive local degradation (relevant to Section ‘Design and functionality of vascular stent materials’).
Myocardial Patch	A biomaterial patch used for the repair of infarcted myocardial tissue. It typically possesses mechanical support and electrical conductivity (*e.g.*, incorporating conductive polymers), which promotes synchronized contraction of cardiomyocytes, reduces inflammation, and enhances vascularization to restore the function of damaged myocardium (detailed in Section ‘Development and optimization of myocardial patch materials’).
Wnt/β-Catenin Pathway	An important cellular signaling pathway. As mentioned in the manuscript, some biomaterials (*e.g.*, lithium-doped silicate microparticles) can activate this pathway to regulate cell proliferation and differentiation, thereby facilitating cardiovascular tissue regeneration (*e.g.*, chondrogenesis and osteogenesis-related repair, referenced in Section ‘Signaling mechanisms of biomaterials in tissue regeneration’).
Extracellular Vesicles-in-Hydrogel	A composite system that combines extracellular vesicles with hydrogels. It enables the tunable and sustained release of extracellular vesicles, integrating the adhesiveness of hydrogels with the trophic effects of extracellular vesicles. It is used to modulate post-injury inflammation and fibrosis, assisting in cardiovascular tissue repair (introduced in Section ‘The potential of regenerative medicine in cardiovascular diseases’).
Polyhydroxyalkanoates	A class of biodegradable polyester-based synthetic biomaterials with tunable mechanical properties and degradation rates, as well as good biocompatibility. They are applied in cardiovascular tissue engineering, such as for heart valve scaffolds and vascular grafts, and are particularly suitable for growth-related repair needs in pediatric patients (discussed in Sections ‘The potential of regenerative medicine in cardiovascular diseases’, ‘Applications in heart valve repair and regeneration’).

**Table A2:** Material abbreviation glossary.

Abbreviation	English full name	Core applications in the manuscript
PLA	Poly (lactic acid)	Vascular stents, myocardial patch scaffolds, composite scaffold matrices
PCL	Poly(*ɛ*-caprolactone)	Electrospun scaffolds, biodegradable vascular stents, mechanical reinforcement phase in composite scaffolds.
PEG	Polyethylene glycol	Hydrogel matrices, drug delivery carriers, immunomodulatory materials.
PHAs	Polyhydroxyalkanoates	Tissue-engineered vascular grafts (TEVGs), pediatric cardiovascular repair materials.
PDO	Polydioxanone	Electrospun scaffolds, myocardial patches, vascular repair scaffolds.
MOFs	Metal–organic frameworks	Biocompatibility-enhancing materials, antimicrobial/anti-inflammatory coatings.
ECM	Extracellular matrix	Natural scaffold matrices, functional modification components for biomaterials.
TEVGs	Tissue-engineered vascular grafts	Vascular repair and replacement materials.

## Additional Information and Declarations

### Data Availability

The following information was supplied regarding data availability: This is a literature review.
